# 

**Published:** 1873-08

**Authors:** 


### INDEX.

**A**

Abbott, F. W., M. D. Glasses called "London Smoke" are better for Shielding the Eyes. Translated from Les Annals D'Oculistique ... 210
Ablation of Complete Staphyloma of the Cornea. By L. DeWecker. Translated by H. Norton, M. D. ... 1
Abstract of the Proceedings of the Buffalo Medical Association ... 121, 363. 426, 457
A Case of Aneurism of the Aorta opening into the trachea ... 462
A Case of Colles Fracture with Luxation of the Ulna. By Chas. D. Buckley, M. D. ... 89
A Case of Renal Tumor. By Dr. Denman 50
A Case Showing the Danger of Error in Diagnosis. By Prof. M. G. Potter, M. D. ... 83
Address before the Graduating Class of the Buffalo Medical College. By Joseph Warren, Esq., Feb'y 24, 1874 ... 306
Albany County Medical Society, Annual Meeting. Reported by F. C. Curtis, M. D. ... 171
Albany County Medical Society, Semi-Monthly Meetings. Reported by F. C. Curtis, M. D. ... 5, 245, 300, 373, 415, 449
Albuminuria Complicating Pregnancy ... 135
Alumni Association of Albany Medical College. ... 237
A Medical License for Ladies ... 305
American Medical Association, Meeting of 353
" " " Report of Meeting ... 433
Amputation at the Shoulder Joint. Remarks and Cases. By J. F. Miner, M. D. ... 81
Anæsthesia, Death from ... 23
Aneurism, New Operation for ... 155
A Paper read before the Montgomery Co,, Ill., Medical Society. By T. D. Washburn, M. D ... 41
Appointment ... 155
Appropriation for the U. S. Medical Museum and Library ... 235
A Report of Five Cases of Colles Fracture, Treated after the plan of Prof. E. M. Moore, M. D. By B. L. Hovey, M D... 47
Atropine as an Antidote to Opium ... 67

**B**

Bigelow, H. R., M. D Recent Investigations into the Physiological Functions of the Brain ... 405
Books and Pamphlets Received, 40, 80, 120, 160, 200, 240, 280. 320, 359, 400, 440, 475
Bowen. G. W., M. D. Case of Fracture of both Bones of the Leg, treated in Wood's Hammock Splint ... 342
Brachial Plexus, Injury of, resulting in Gangrene of the Arm and fore arm. By C. C. F. Gay, M. D. ... 17
Brain, Gunshot wound of. By P. W. Van Peyma. M. D ... 164
Bright's Disease. The Pathology and Diagnosis of Acute ... 62
Brush, E. N. Items and Remarks by..72, 113
" " Report of Clinical Remarks by J. F. Miner, M. D ... 127
Buckley, Chas. D., M. D. A Case of Colles Fracture with Luxation of the Ulna. 89
Buffalo City Dispensary ... 274
" Medical Association, Abstract of Proceedings of ... 121, 363, 426, 457
" Medical College. Twenty-Eighth Annual Commencement ... 273
" Mortality Statistics ... 3[illegible]4
" State Insane Asylum ... 267
" Water Supply ... 109
Butler, S. W., M. D. Obituary ... 235

**C**

Cancer of the Breast, Remarks on ... 149
Carbonate of Ammonia in Scarlet Fover . 60
Cerebral Pathology, Experimental Researches in ... 215
Cerebro Spinal Menengitis, a mild case followed by Blindness ... 466
Cholera, Asiatic, History of ... 25
" A Certain sign for the recognition in early stages ... 29
Chloroform and Ether ... 198
Circular to the Medical Profession ... 218
Classification of Skin Diseases. A Synopsis of Essay on. By J. W. Southworth, M. D ... 166
Clinical Remarks upon Surgical Cases in the Buffalo Hospital of the Sisters of Charity. By J. F. Miner M. D ... 127
Colegrove, Bela H., M. D. Obituary ... 315
Colvin, D , M. D., Pelvic Cellulitis ... 441
Commencement, Twenty-Eighth Annual of the Buffalo Medical College ... 273
Concentrated Palatable Powders. By J. W. Southworth, M. D ... 264
**CORRESPONDENCE.—**
Action of the Monroe County Medical Society in relation to the Publication of the State Society Transactions 131
Fracture of the Cervix Femoris ... 180
Procedentia Uteri ... 132
Croton Chloral ... 378
" " Hydrate ... 214
Curtis, F. C , M. D. Report of Transactions of the Albany County Medical Society ... 5, 171, 245, 300, 373, 415, 449

**D**

Defective Drainage ... 275, 381
Denman, Dr. A Case of Renal Tumor... 50
Dictionaries ... 236
Distribution of the Medical and Surgical History of the War ... 33

Doctor needing Reconstruction.......... 317
Dysentery, Chronic. On the Treatment of 70
" " On the Use of Chlorate of Potash and Glycerine for Ulcerations in.......... 91

**E**

Earth. The Application of, in Surgery... 106
Eastman, Prof. Sanford, M. D. Obituary. 232
Editorial Change.......... 314
Election of Officers..........155, 355
Electricity, Medical.......... 186
Electricity, Notes of Cases Treated by. By G. W. Frion, M. D.......... 15
Electro-Therapeutics, Observations in. By A. D. Rockwell, M. D.......... 333
Epilepsy, Nitrite of Amyl in.......... 190
Errata.......... 75
Esmarch's Bloodless Operations.......... 270
Experimental Researches in Cerebral Pathology.......... 215

**F**

Fractures, Hints on the Treatment of. By J. F. Woods, M. D.......... 201
Fracture of both bones of the Leg, treated in Wood's Hammock Splint. By G. W. Bowen, M. D.......... 342
Flint, Prof. Austin, M. D., on the Diffusion of Typhoid Fever by means of Drinking Water.......... 281
Frion, G. W., M. D. Notes of Cases treated by Electrrcity.......... 15

**G**

Gastrotomy Successfully performed in a Case of Extra-Uterine Pregnancy.......... 30
Gay, C. C. F., M. D. Injury of the Brachial Plexus, resulting in Gangrene of the Arm and Fore-arm.......... 17
Glasses called "London Smoke" are better for shielding the Eye. Translated from Les Annales D'Oculistique. By F. W. Abbott, M. D.......... 210
Guarana.......... 355

**H**

Hairy Man.......... 318
Hamilton Allan, McLane, M. D. The Nervous Diseases of Women.......... 401
Hamilton, Prof. F. H,. M. D. Ingrowing Toe-Nail.......... 361
Hamilton, Prof. F. H., M. D. And the N. Y. Commissioners of Public Charities and Corrections.......... 437
Hard on the Coroners.......... 236
Hints on the Treatment of Fractures. By J. F. Woods, M. D.......... 201
Holston, Dr. John F., Sr. Obituary.......... 396
Hovey, B. L., M. D. A Report of Five Cases of Colles Fracture.......... 47
Hypodermic Syringe. A New.......... 182

**I**

Infantile Paralysis.......... 59
Ingrowing Toe Nail. By Prof. F. H. Hamilton, M. D.......... 361
Immediate Compression of the Common Illiac.......... 227
Insane Asylums, The Regulation of.......... 305
Intermittent Fever, Development of by Obstructed Drains.......... 381
Intra-Recto-Abdominal Exploration.......... 19
Introductory Lecture.......... 155
Intussusception, Successful Abdominal Section for.......... 351
Items in Brief.......... 33
Items and Remarks. By. E. N. Brush. 72, 123, 395, 471

**K**

Knowles, J., M. D. Case of Splenic Abscess.......... 161

**L**

Laws of New York Regulating the Office of Coroner.......... 71
Law Regulating the Practice of Medicine and Surgery.......... 436
Life Insurance, Report on.......... 348
Light, Sanitary Value of.......... 31
Livingston, Dr. Alfred T., Appointment of 155

**M**

Malignant Disease of the Uterus. By H. D. Vosburgh, M. D.......... 207
Medical and Surgical History of the War, Distribution of the.......... 33
Medical Education, The Standard of in America.......... 393
Medical Electricity.......... 186
Mercury, The Oleate of.......... 22
Miner, J. F., M. D. Amputation at the Shoulder Joint.......... 81
Miner, J. F., M. D. Clinical Remarks upon Surgical Cases in the Buffalo Hospital of the Sisters of Charity.......... 127
Moonshine in Medicine.......... 468
Morbid Impulses.......... 383

**N**

Nervous Diseases of Women. By Allan McLane Hamilton, M. D.......... 401
Nervous Diseases of Women.......... 104
" " Syphilitic Patients, the Treatment of.......... 55
" " The Chicago Journal of.......... 355
Neurasthenia and its Treatment.......... 391
New Journal..........236–275
Niagara River Water. Is it healthy?.......... 72
Nitrite of Amyl in Epilepsy.......... 190
Norton, H., M. D., Ablation of Complete Staphyloma of the Cornea. By L. Dr. Wecker. Translation.......... 1
Notes of cases Treated by Electricity. By G. W. Frion, M. D.......... 15
Notice.......... 236

**O**

**OBITUARY.—**
Prof. Sandford Eastman, M. D. 232
S. W. Butler, M. D.......... 285
Bela H. Colegrove, M. D.......... 315
John F. Holston, Sr., M. D.......... 395
Prof. Jas McNaughton, M. D.. 471
O'Brien, E. C. W., M. D. Re-appointment of as Health Physician.. 236
Observations in the Treatment of Yellow Fever.......... 143
Old Eyes Made New. By Charles E. Robertson, M. D.......... 241
Oleate of Mercury.......... 22
On the Diffusion of Typhoid Fever by means of Drinking Water. By Prof. Austin Flint, M. D.......... 2[illegible]1
Opium, Atropine as an Antidote.......... 67
Opium Poisoning, Use of Atropia.......... 187
Ovarian Tumor removed by Enucleation. 52
Ovariotomy, A Case of,.......... 183
" By Enucleation, ..........19–223–2[illegible]9
" Under Difficulties,.......... 3[illegible]9

**P**

Paralysis, Infantile, ........ 50
Paralysis of the hand and forearm from Esmarch's bloodless method ........ 464
Peabody, Chas. H., A. M., M. D. Report of a case of fatal Respiratory obstruction from softened Mediastinal Glands. A Case of Extraordinary Discharge of Lumbrici ........ 447
Pelvic Cellulitis. By D. Colvin, M. D ........ 441
Periodicals and Pamphlets ........ 39
Periodical Literature, ........ 159
Phosphorus, A New Solvent for ........ 216
Physiological Functions of the Brain. Recent Investigations into. By H. R. Bigelow, M. D. ........ 405
Potter, Prof. Milton, G., M. D. A Case Showing the Danger of Error in Diognosis 83
Pregnancy, Extra Uterine, A case of ........ 30
Pulmonary Consumption, The Importance and Dangers of Rest in ........ 272
Puncturing the Bladder above the Pubis. By W. C. Raymond, M. D. ........ 88

**R**

Rank of the Medical Corps of the U. S. Army ........ 195
Rank of the Medical Corps of the U. S. Army. Passage of a Bill to Regulate the ........ 436, 470
Raymond, W. C., M. D. Puncturing the Bladder above the Pubis 88
Re-appointment, ........ 236
Recovery from Ruptured Uterus and Protrusion of the Intestine ........ 108
Removal of the Superior Maxilla, ........ 24
Renal Lesions following Cerebral Hemorrhage ........ 467
Renal Tumor, A case of. By Dr. Denman 50

**REVIEWS.—**

A Guide to Urinary Analysis. By Henry G. Piffard, M. D. ........ 76
A Hand-book of Medical Electricity. By Herbert Tibbitts, M. D. ........ 35
A Hand book of the Theory and Practice of Medicine By T. F. Roberts, M B., B. Sc. ........ 276
A Manual of Medical Jurisprudence. By Alfred Swaine Taylor, M. D. ........ 156
A Manual of Midwifery. By Dr. Karl Schrœder, ........ 240
A Manual of Toxicology. By John J. Reese, M. D. ........ 473
A Practical Treatise on Diseases of the Ear. By D. B. St. John Roosa, M. A., M. D. ........ 199
A Practical Treatise on Diseases of Children. By Drs. Meigs and Pepper ........ 318
A Report on the Origin and Therapeutic Properties of Cundurango. By W. S. W. Ruschenberger M. D. ........ 26
A System of Midwifery. By Wm. Leishman, M. D. ........ 239
A Treatise on the Principles and Practice of Medicine. By Austin Flint, M. D ........ 39
A Treatise on Diseases of the Eye By J. Sœlberg Wells, F. R. C. S. 277
A Treatise on Diseases and Injuries of the Eye. By Dr. Carl Stellwag ........ 238
An Introduction to The Study of Clinical Medicine ........ 319
Annual Report of the Supervising Surgeon of the U. S. Marine Hospital Service ........ 359
Barnes on the Diseases of Women. 399
Bellamy's Guide to Surgical Anatomy ........ 358
Chemistry, General, Medical and Pharmaceutical. By Jno. Attfield Ph. D., F. C. S. ........ 79
Chemistry, Inorganic and Organic By Chas. L. Bloxam, ........ 156
Clinical Electro-Therapeutics Medical and Surgical. By Allan McLane Hamilton, M. D. ........ 159
Clinical Researches in Electro Surgery By Drs. Beard & Rockwell. 219
Contributions to Practical Surgery By Geo. W. Norris, M. D. ........ 78
Dental Caries and its Causes. By Drs. Leber & Rottenstein ........ 35
Dunglison's Medical Dictionary, ........ 356
Family Thermometry. By Edward Seguin, M. D ........ 38
Galvano Therapeutics. By David Prince, M. D ........ 472
Griffith's Formulary ........ 356
Gunshot Wounds of the Abdomen By Eugene Peugnet, M. D ........ 357
Hand Book of Physiology. By Wm. Senhouse Kirkes, M D ........ 157
History of the American Ambulance in Paris ........ 397
Insanity and its Relations to Crime By Wm. A. Hammond, M. D., ........ 116
Introduction to Physical Measurements. By Dr. F. Kolrausch ........ 472
Lacerations or the Female Perineum and Vesico-vaginal Fistula. By D. Hayes Agnew, M. D., ........ 277
Lectures on Clinical Medicine. By Trousseau, ........ 237
Lectures on Clinical Uses of Electricity. By J Russell Reynolds, M. D., F. R. S ........ 472
Lectures on Diseases and Injuries of the Ear. By W. B. Dalby, F. R. C. S ........ 199
Manual of Chemical Analysis as applied to the Examination of Medical Chemicals. By F Hoffman, Ph D ........ 77
Mineral Springs of the U. S. and Canada. By Geo E. Walton, M. D. 158
On the Cerebral Convolutions of Man By Alexander Ecker ........ 276
On the Mechanical Treatment of Diseases of the Hip-joint. By Chas. F. Taylor, M. D. ........ 200
Operative Manual, Ligation of Arteries By L. H. Farabeuf Translated by John D Jackson, M. D ........ 474
Pharmaceutical Lexicon By H. V. Sweringen ........ 158
Physicians' Hand Book, ........ 238
Physicians' Visit ng List ........ 238
Report of Columbia Hospital for Women, ........ 157
Sex in Education. By Edward H. Clark, M. D., ........ 278
Skin Diseases. Their Pathology, Diagnosis and Treatment. By Tilbury Fox, M. D., ........ 79
Text Book of Physiology. By Jno. Hughes Bennet, M. D , ........ 77
The Mechanism of the Ossicles of the Ear. By H. Helmholtz, ........ 279

The Passions in their Relation to Health and Disease. Translated by H F. Damon, M. D., ........ 38
The Principles and Practice of Surgery. By Frank H. Hamilton, M. D., ........ 76
The Puerperal Diseases. By Fordyce Barker, M. D., ........ 398
The Science of Homeœpathy. By Charles J. Hempel, M. D ........ 474
The Sphygmograph. By Edgar Holden, M. D., ........ 358
Robertson, Charles A., M. D. Old Eyes made New ........ 241
Rockwell, A. D., M. D. Observations on Electro-Therapentics ........ 333
Rubber Bands as aids to Stethoscopic Examinations. By J. W. Southworth, M. D. 49
Rules for the Management of Infants ........ 101

S

Scarlet Fever, Carbonate of Ammonia in, 60
Septicæmia The Controversy on ........ 5[illegible]
Shoulder Joint, Amputation at. By J. F. Miner, M. D ........ 81
Skull, A Case of Loss of nearly half the Bones of the, ........ 95
Southworth, J. W., M. D. A Synopsis of Essay on Classification of Skin Diseases ........ 166
" " Concentrated and Palatable Powders ........ 264
" " Rubber Bands as Aids to Stethoscopic examinations ........ 49
Sphygmograph. A Practical, ........ 219
Splenic Abscess, A Cure of. By J. Knowles, M. D., ........ 161
Staphyloma of the Cornea, Ablation of. By L. DeWecker, Translated by H. Norton, M. D. ........ 1
State Medical Society. Meeting of, ...236—275
" " Publication of Transactions, of The, ........ 155
Strychnine Poisoning, ........ 221
Superior Maxilla, Removal of, ........ 24
Syphilis, Mercury in ........ 350
Syphilitic Patients. The Treatment of Nervous Diseases of, ........ 55

T

Tatooing the Cornea ........ 57
The Standard of Medical Education in America ........ 393
Thoughts on Medical Progress. By T. D. Washburn, M. D ........ 294
Toe Nail, Ingrowing. By Prof. Frank H. Hamilton, M. D ........ 361
Transfusion, ........ 344
Typhoid Fever. Foul Wells the Cause of. 90
" " On the Diffusion of, by means of Drinking Water. By Prof. Austin Flint, M. D. 281

U

Uterine Flexions. The Treatment of. By Ely Van DeWarker, M. D. ........ 321
Uterus, Malignant Disease of the. By H. D Vosburgh, M. D ........ 207
Uterus, Ruptured. A case of Recovery from ........ 108

V

Van DeWarker. Ely, M. D. The Treatment of Uterine Flexions ........ 321
Van Peyma, P. W., M. D. Gunshot Wound of the Brain, ........ 164
Vascular Nævi, The Treatment of by Galvanic Cantery ........ 387
Volume Thirteen ........ 32, 470
Vosburgh, H. D., M. D. Malignant Disease of the Uterus ........ 207

W

Warren. Joseph, Esq. Address before the Graduating Class of the Buffalo Medical College. Feb'y 24, 1874. ........ 306
Washburn, T. D., M. D. A Paper read before the Montgomery Co., Ill., Medical Society ........ 41
Washburn, T. D., M. D. Thoughts on Medical Progress, ........ 2[illegible]4
Water, Rendering Hard, Soft ........ 100
" The Supply of Buffalo ........ 109
Woods, J. F, M. D. Hints on The Treatment of Fractures ........ 201

### Books and Pamphlets Received.

Skin Diseases: Their Description, Pathology, Diagnosis and Treatment. By Tilbury Fox, M. D., Lond., etc. Second American, from Third London Edition, Re-written and Enlarged. New York: Wm. Wood & Co., 1873. Buffalo: H. H. Otis.

Pharmaceutical Lexicon: A Dictionary of Pharmaceutical Science, containing a concise explanation of the various subjects and terms of Pharmacy, with appropriate Selections from the Collateral Sciences. By H. V. Sweringen, Member American Pharmaceutical Association, etc. Philadelphia: Lindsay & Blakiston, 1873. Buffalo: T. Butler & Son. By Subscription only. Price Sheep $7.00, Cloth $6.00.

Contributions to Practical Surgery. By Geo. W. Norris, M. D. Philadelphia: Lindsay & Blakiston, 1873. Buffalo: T. Butler & Son. Cloth, Price $4.00.

Chemistry: General, Medical, and Pharmaceutical. Including the Chemistry of the U. S. Pharmacopœia. A Manual of the General Principles of the Science and their Applications to Medicine and Pharmacy. By John Attfield, Ph. D., F. C. S., Fifth Edition. Philadelphia: Henry C. Lea, 1873. Buffalo: T. Butler & Son.

On the Treatment of Diseases of the Skin: With an Analysis of eleven thousand consecutive cases. By Dr. McCall Anderson. Philadelphia: Henry C. Lea, 1873. Buffalo: T. Butler & Son.

An Introduction to the Study of Clinical Medicine: Being a Guide to the Investigation of Disease, for the use of Students. By Octavius Sturges, M. D., Cantab., etc. Philadelphia: Henry C. Lea, 1873. Buffalo: T. Butler & Son.

The Mechanism of the Ossicles of the Ear and Membrana Tympani. By H. Helmholtz. Translated from the German by Albert H. Buck and Normand Smith, of New York. New York: Wm. Wood & Co., 1873.

On Ovariotomy. By J. Marion Sims, M. D. Reprinted from N. Y. Medical Journal, Dec. 1872 and April 1873. New York: D. Appleton & Co., 1873.

The Etiology and Indications for Treatment of Irregular Uterine Action During Labor. By Wm. T. Lusk, M. D. Reprinted from New York Medical Journal, June 1873. New York: D. Appleton & Co., 1873.

Thirtieth Annual Report of the Managers of the State Lunatic Asylum, Utica, N. Y., for the year 1872.

An Eye Case in Court. By C. A. Robertson, A. M., M. D, Albany, 1873.

A Report of the Rochester City Hospital for 1872.

Ophthalmic and Aural Surgery Reports. By Julian J. Chisholm, M. D. Baltimore, 1873.

